# The risk of interstitial lung disease in psoriatic arthritis versus psoriasis: a retrospective nationwide database analysis (2014–24)

**DOI:** 10.1093/rap/rkaf059

**Published:** 2025-05-27

**Authors:** Andrew Engel, Christopher Xie, Ross Summer, Giorgos Loizidis

**Affiliations:** Division of Pulmonary, Allergy and Critical Care and the Center for Translational Medicine, Department of Medicine, The Jane & Leonard Korman Respiratory Institute, Thomas Jefferson University, Philadelphia, PA, USA; Division of Pulmonary, Allergy and Critical Care and the Center for Translational Medicine, Department of Medicine, The Jane & Leonard Korman Respiratory Institute, Thomas Jefferson University, Philadelphia, PA, USA; Division of Pulmonary, Allergy and Critical Care and the Center for Translational Medicine, Department of Medicine, The Jane & Leonard Korman Respiratory Institute, Thomas Jefferson University, Philadelphia, PA, USA; Division of Pulmonary, Allergy and Critical Care and the Center for Translational Medicine, Department of Medicine, The Jane & Leonard Korman Respiratory Institute, Thomas Jefferson University, Philadelphia, PA, USA; Department of Medicine, Thomas Jefferson University, Philadelphia, PA, USA

**Keywords:** interstitial lung disease, psoriasis, PsA

## Abstract

**Objectives:**

Psoriasis (PsO) is a systemic autoimmune disease primarily characterized by erythematous plaques on the skin. While extra-dermal manifestations like psoriatic arthritis (PsA) are well recognized, data linking PsO to interstitial lung disease (ILD) remain limited. This study aimed to evaluate whether patients with PsA have a higher risk of developing ILD compared with patients with PsO.

**Methods:**

A retrospective analysis of the TriNetX US database (2014–24) was performed. Adult patients with PsO or PsA treated with systemic immunosuppressive medications were included, excluding those with other autoimmune diseases. ILD risk in PsO and PsA cohorts was compared with a reference population without autoimmune disease. Propensity score matching (PSM) adjusted for age, sex, race, BMI, smoking status and medications known to cause ILD was performed. Baseline immunosuppressive therapies were included in the PSM when comparing PsO and PsA. Statistical significance was determined using the χ^2^ test of independence.

**Results:**

After PSM, PsA patients (*n* = 13 168) had a significantly higher ILD risk compared with the general population (*n* = 13 168) (risk ratio [RR] 1.94; 95% CI 1.29–2.92; *P* = 0.0011). PsO patients (*n* = 24 039) showed no significant difference in ILD risk compared with controls (*n* = 23 786) (RR 0.79; 95% CI 0.57–1.08; *P* = 0.14). PsA (*n* = 13 838) exhibited an over 1.5 times increase in ILD risk compared with PsO (*n* = 13 842) (RR 1.52; 95% CI 1.06–2.20; *P* = 0.0226).

**Conclusions:**

PsA was associated with a significantly higher likelihood of developing ILD compared with PsO without inflammatory arthritis. These findings underscore the importance of respiratory monitoring in PsA and highlight the need for further studies.

Key messages

- Patients with PsA exhibited nearly twice the risk of interstitial lung disease compared to matched general population controls.
- PsA conferred an over 1.5-fold higher risk of interstitial lung disease compared to psoriasis alone.
- This highlights the potential role of inflammatory arthritis in increasing pulmonary involvement.

## Introduction

Psoriasis (PsO) is a systemic autoimmune disease primarily characterized by erythematous plaques on the skin, commonly affecting the scalp, elbows, knees and back. Approximately 30% of individuals with PsO develop PsA, an inflammatory arthritis influenced by risk factors such as older age, female sex, obesity and severe skin/nail involvement [1–3]. Patients with PsA also exhibit a higher prevalence of cardiometabolic comorbidities, including metabolic syndrome, type 2 diabetes and hypertension [4].

Recent findings suggest that psoriatic disease may predispose to pulmonary complications, notably interstitial lung disease (ILD) [5–10]. In one retrospective study of 387 patients with PsA, nearly half reported extra-articular manifestations, with approximately 1% diagnosed with ILD—a higher prevalence than observed in the general population [10, 11]. Moreover, the radiologic and histologic patterns in those with psoriasis who have ILD often align with nonspecific interstitial pneumonia (NSIP), which is similarly seen in other autoimmune conditions such as rheumatoid arthritis (although the most common form is usual interstitial pneumonia [UIP]) and Sjögren’s syndrome [7, 12–14]. In addition to retrospective series and clinical case reports, a recent large registry-based epidemiologic study of 10 919 PsA patients in Nordic rheumatology registries also identified an increased risk of ILD compared with controls [15].

Despite these observations, additional large-scale, population-based investigations are needed to clarify whether PsA confers a greater risk of ILD compared with psoriasis without inflammatory joint involvement. In this retrospective analysis of an international database, we compare ILD risk in patients with PsA versus those with PsO. We hypothesized that individuals with psoriasis and PsA are at increased risk of ILD compared to patients with PsO alone.

## Methods

We conducted our investigation utilizing the TriNetX US Collaborative Network, which aggregates de-identified electronic health records (EHRs) from over 60 healthcare organizations. The frequency of health record updates varies by institution, occurring anywhere from daily to bi-monthly. TriNetX complies with ISO 27001:2013 standards and the HIPAA Security Rule, ensuring robust data security. Our analysis focused on US data spanning from 2014 to 2024, and data collection was completed on 16 November 2024. Due to the de-identified nature of our data, Institutional Review Board approval was not required.

### Cohort formation

**Figure 1.**
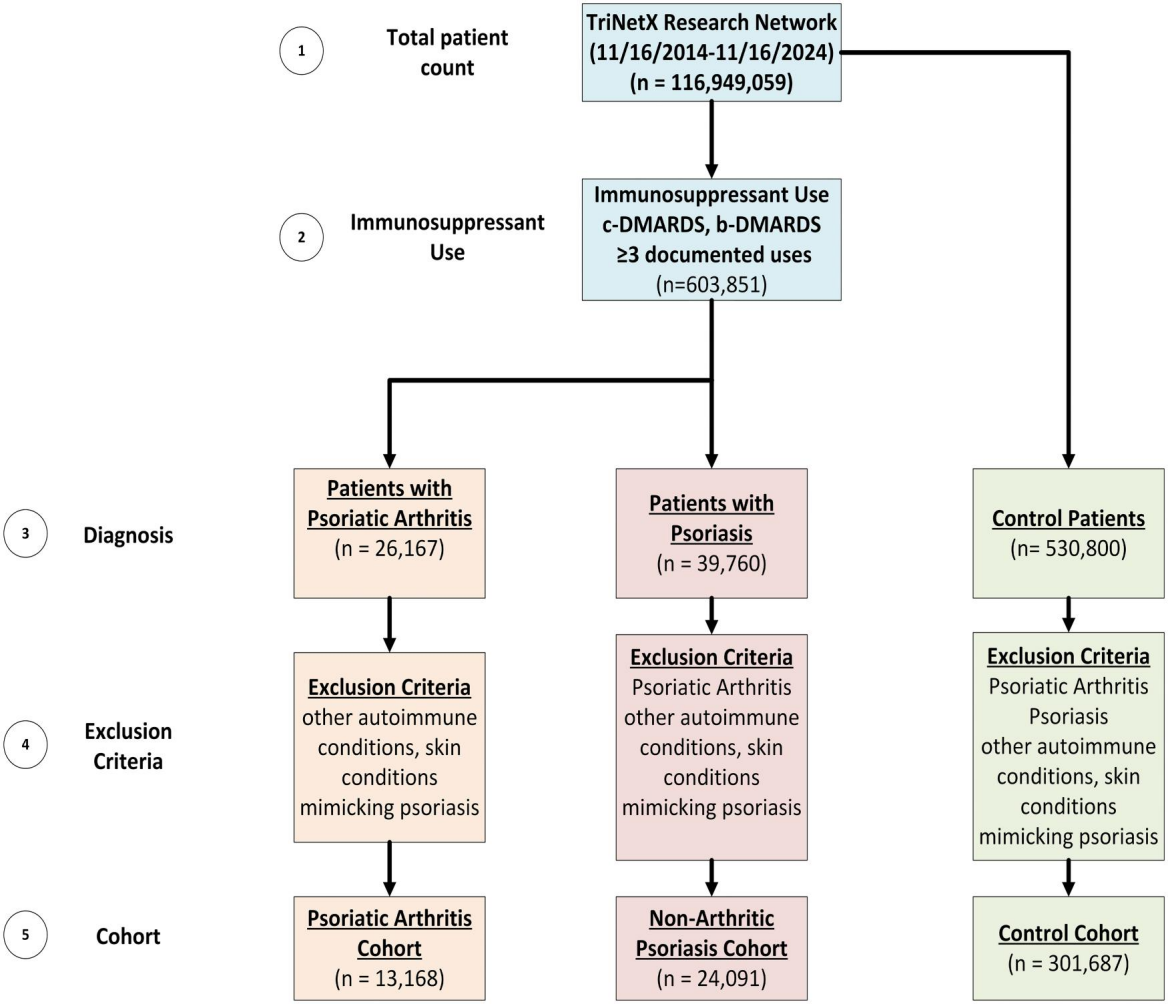
Flow diagram illustrating the selection process for the PsA, non-arthritic psoriasis and control cohorts from the TriNetX Research Network database

We identified three distinct study cohorts—PsA, PsO, and a general control population—using ICD-10, RxNorm, TriNetX-curated, and LOINC codes for relevant diagnoses, medications, comorbidities, and laboratory tests (see Supplementary Table S1, available at *Rheumatology Advances in Practice* online, for details). The PsO cohort consisted of patients diagnosed with psoriasis (ICD-10 code L40) but without any diagnosis of PsA (ICD-10 code L40.50) or other inflammatory arthritis. The PsA cohort included patients specifically diagnosed with PsA. Both PsO and PsA cohorts were required to have at least three documented prescriptions for immunosuppressive agents (either conventional or biologic DMARDs) to ensure active treatment of psoriatic disease. The control cohort included individuals coded with ICD‐10 Z00.6 (encounter for examination for normal comparison and control in clinical research) who had no recorded history of autoimmune disease. A detailed summary of the cohort formation is presented in Fig. 1.

To preserve cohort specificity, we excluded patients with rheumatoid arthritis, systemic lupus erythematosus, dermatomyositis, giant cell arteritis, ANCA vasculitis, Sjögren’s syndrome, polymyalgia rheumatica, systemic sclerosis, IgG4‐related disease, malignancy, ankylosing spondylitis, inflammatory polyarthropathy, Crohn’s disease, ulcerative colitis, pemphigus, atopic dermatitis, multiple sclerosis, sarcoidosis and large vessel vasculitis. We also excluded individuals testing positive for rheumatoid factor, anti-CCP antibody or anti-SSA, in order to remove overlapping syndromes that might spuriously elevate ILD risk without a definitive rheumatologic diagnosis. To minimize misclassification of psoriasis, we further excluded skin conditions that resemble psoriasis (e.g. lichen sclerosus et atrophicus, hidradenitis suppurativa, nummular dermatitis, seborrheic dermatitis and lichen simplex chronicus).

### Index event and time window

The index event (IE) was defined as the first recorded PsA or PsO diagnosis in the EHR. At that same date, patients were required to be on psoriatic disease-specific immunosuppressive therapy (e.g. methotrexate, TNF inhibitors, secukinumab, ixekizumab or apremilast; see Supplementary Table S1, available at *Rheumatology Advances in Practice* online) to confirm active treatment. The primary outcome was a new diagnosis of ILD, identified via ICD‐10 codes for unspecified interstitial pulmonary disease (J84.9). ILD events recorded from 1 day to 5 years following the IE were included; any patient with a prior ILD diagnosis was excluded before defining the IE.

### Covariate and propensity score matching

After defining cohorts, IEs, outcomes and potential confounders, a logistic regression model estimated the probability (propensity score) that each patient belonged to the comparison cohort, given their baseline covariates. Using a greedy nearest‐neighbour algorithm and a 0.1 pooled standard deviation caliper, we matched patients from the smaller cohort to those in the larger cohort with similar propensity scores.

Propensity score matching (PSM) was conducted for all three comparisons—PsA versus the general population, PsO versus the general population, and PsA versus PsO—to account for potential confounders. The following key covariates were included: age, race, sex, chronic obstructive pulmonary disease (COPD), smoking, pneumoconiosis and medications known to cause ILD, such as amiodarone. Additionally, gastroesophageal reflux disease (GERD) and obstructive sleep apnoea (OSA) were included because both conditions are associated with metabolic syndrome and obesity—factors linked to the severity of psoriatic disease [16]—and are recognized comorbidities in ILD implicated in its pathogenesis and progression [17, 18]. Because TriNetX collects BMI primarily in categorical ranges rather than continuous values, we matched participants based on BMI categories (e.g. 18–24.9, 25–29.9, 30–39.9, ≥40 kg/m^2^). This approach minimized data loss due to imputation or casewise deletion, thereby preserving both sample size and statistical power. A standardized mean difference (SMD) of less than 0.1 was considered indicative of adequate covariate balance after PSM, following established methodological guidelines [19]. A complete list of covariates used in the PSM is provided in Supplementary Table S2, available at *Rheumatology Advances in Practice* online.

### Statistical analysis

All analyses were conducted within the TriNetX Advanced Analytics Platform. This system computes risk ratios (RRs), odds ratios and their corresponding 95% CIs, with statistical significance for risk comparisons assessed using two‐sided *P* < 0.05 (χ^2^ tests). In each matched comparison, we evaluated the incidence of new‐onset ILD during the 1‐day to 5‐year follow‐up period, thereby comparing ILD risk in PsA versus the general population, PsO versus the general population, and PsA versus PsO.

### Negative control analysis

To assess residual confounding, two negative control outcomes—injury (ICD-10 T14.90XA) and Tdap vaccination (CPT 90715)—were used. The chosen negative controls are previously validated proxies to detect potential confounding related to healthcare access or health behaviours and are unrelated to the underlying pathology of ILD, PsA or PsO [20–22].

## Results

### PsA versus general population

#### Baseline characteristics before matching

Initially, 13 168 patients with PsA were compared with 301 687 individuals from the general population (Table 1). PsA patients were younger on average (49.9 ± 13.6 vs. 55.8 ± 22.8 years, *P *< 0.001) and had a higher mean BMI (32.5 ± 7.8 vs. 29.0 ± 7.1, *P *< 0.001). Comorbidities such as GERD, smoking and COPD were also more common in the PsA cohort.

**Table 1.** Baseline characteristics of PsA versus general population before and after propensity score matching

	Unadjusted baseline characteristics				Adjusted baseline characteristics			
	PsA	General population	*P*-value	Std diff.	PsA	General population	*P*-value	Std diff.
	(*n* = 13 168)	(*n* = 301 687)			(*n* = 13 168)	(*n* = 13 168)		
	Mean ± SD	Mean ± SD			Mean ± SD	Mean ± SD		
Age at index	49.9 ± 13.6	55.8 ± 22.8	<0.0001	0.321	49.8 ± 13.6	50.1 ± 14.1	0.075	0.022
BMI	32.5 ± 7.8	29.0 ± 7.1	<0.0001	0.461	32.5 ± 7.8	32.1 ± 7.6	<0.001	0.055

	Unadjusted # patients (% of cohort)				Adjusted # patients (% of cohort)			

White	10 831 (82.3%)	208 652 (69.2%)	<0.0001	0.309	10 831 (82.3%)	10 916 (82.9%)	0.167	0.017
Female	7495 (56.9%)	151 638 (50.3%)	<0.0001	0.134	7495 (56.9%)	7486 (56.8%)	0.911	0.001
Male	5346 (40.6%)	142 043 (47.1%)	<0.0001	0.151	5346 (40.6%)	5491 (41.7%)	0.069	0.022
Black or African American	395 (3.0%)	34 615 (11.5%)	<0.0001	0.332	395 (3.0%)	419 (3.2%)	0.393	0.011
Asian	387 (2.9%)	10 045 (3.3%)	0.014	0.022	387 (2.9%)	376 (2.9%)	0.686	0.005

Medical history	Unadjusted # patients (% of cohort)				Adjusted # patients (% of cohort)			

Gastro-esophageal reflux disease	1548 (11.8%)	45 547 (15.1%)	<0.0001	0.098	1548 (11.8%)	1535 (11.7%)	0.803	0.003
Nicotine dependence	664 (5.0%)	22 193 (7.4%)	<0.0001	0.096	664 (5.0%)	619 (4.7%)	0.198	0.016
Personal history of nicotine dependence	532 (4.0%)	49 714 (16.5%)	<0.0001	0.419	532 (4.0%)	511 (3.9%)	0.507	0.008
Sleep apnoea	1043 (7.9%)	30 507 (10.1%)	<0.0001	0.077	1043 (7.9%)	977 (7.4%)	0.126	0.019
Other chronic obstructive pulmonary disease	264 (2.0%)	22 818 (7.6%)	<0.0001	0.263	264 (2.0%)	230 (1.7%)	0.123	0.019
Contact with and (suspected) exposure to asbestos	10 (0.1%)	436 (0.1%)	0.041	0.021	10 (0.1%)	10 (0.1%)	1	0
Pneumoconiosis due to dust containing silica	0	17 (0.0%)	0.389	0.011	0	0	–	–

Medications								

Nitrofurantoin	283 (2.1%)	7531 (2.5%)	0.012	0.023	283 (2.1%)	286 (2.2%)	0.899	0.002
Amiodarone	22 (0.2%)	10 103 (3.3%)	<0.0001	0.244	22 (0.2%)	22 (0.2%)	1	0

#### Post‐match comparisons

After one‐to‐one PSM, each cohort included 13 168 patients (Table 1). Baseline variables were well balanced, with SMDs below 0.1 for most covariates. However, PsA patients continued to show a higher mean BMI (32.5 ± 7.8 vs. 32.1 ± 7.6, *P *< 0.001), with a standardized difference of 0.05.

#### Risk of ILD

Within a 1‐day to 5‐year observation window, ILD was diagnosed in 0.52% of PsA patients versus 0.27% in the matched general population. The RR was 1.94 (95% CI: 1.29–2.92; *P *< 0.01), indicating a significantly elevated risk of ILD among PsA patients compared with matched controls (Table 2).

**Table 2.** Results for analyses A, B and C

Analysis A (5 years) Adjusted risk ratio 43 patients in cohort 1 and 41 patients in cohort 2 were excluded from results because they had the outcome prior to the time window.				

PsA (*n* = 13 168)	General population (*n* = 13 168)	Risk ratio	*P-*value*	CI

68 (0.52%)	35 (0.27%)	1.94	<0.01	1.29–2.92
Analysis B (5 years) Adjusted risk ratio 52 patients in cohort 1 and 86 patients in cohort 2 were excluded from results because they had the outcome prior to the time window.				

Psoriasis (*n* = 24 039)	General population (*n* = 23 786)	Risk ratio	*P-*value	CI

67 (0.28%)	85 (0.35%)	0.79	0.14	0.57–1.08
Analysis C (5 years) Adjusted risk ratio 35 patients in cohort 1 and 31 patients in cohort 2 were excluded from results because they had the outcome prior to the time window.				

PsA (*n* = 13 838)	Psoriasis (*n* =13 842)	Risk ratio	*P-*value*	CI

73 (0.53%)	48 (0.35%)	1.52	<0.05	1.06–2.20

### Psoriasis versus general population

#### Baseline characteristics before matching

There were 24 091 patients with psoriasis (PsO) and 301 687 from the general population (Table 3). PsO patients were younger (48.3 ± 18.1 vs. 55.8 ± 22.8 years, *P *< 0.001), more likely to be White (74.2% vs. 69.2%, *P *< 0.001), and had a higher BMI on average (31.0 ± 8.0 vs. 29.0 ± 7.1, *P *< 0.001). Comorbidities such as GERD, COPD and smoking were also more prevalent in the PsO group.

**Table 3.** Baseline characteristics of psoriasis versus general population before and after propensity score matching

	Unadjusted baseline characteristics				Adjusted baseline characteristics			
	Psoriasis	General population	*P*-value	Std diff.	Psoriasis	General population	*P*-value	Std diff.
	(*n* = 24 091)	(*n* = 301 687)			(*n* = 24 091)	(*n* = 24 091)		
	Mean ± SD	Mean ± SD			Mean ± SD	Mean ± SD		
Age at index	48.3 ± 18.1	55.8 ± 22.8	<0.0001	0.365	48.3 ± 18.1	48.5 ± 18.4	0.22	0.011
BMI	31.0 ± 8.0	29.0 ± 7.1	<0.0001	0.264	31.0 ± 8.0	30.8 ± 7.8	0.014	0.032

	Unadjusted # patients (% of cohort)				Adjusted # patients (% of cohort)			

White	17 883 (74.2%)	208 652 (69.2%)	<0.0001	0.113	17 883 (74.2%)	18 014 (74.8%)	0.171	0.012
Female	12 091 (50.2%)	151 638 (50.3%)	0.824	0.001	12 091 (50.2%)	12 046 (50.0%)	0.682	0.004
Male	11 578 (48.1%)	142 043 (47.1%)	0.003	0.02	11 578 (48.1%)	11 646 (48.3%)	0.535	0.006
Black or African American	1390 (5.8%)	34 615 (11.5%)	<0.0001	0.204	1390 (5.8%)	1429 (5.9%)	0.449	0.007
Asian	1028 (4.3%)	10 045 (3.3%)	<0.0001	0.049	1028 (4.3%)	985 (4.1%)	0.328	0.009

Medical history	Unadjusted # patients (% of cohort)				Adjusted # patients (% of cohort)			

Gastro-esophageal reflux disease	2216 (9.2%)	45 547 (15.1%)	<0.0001	0.181	2216 (9.2%)	2141 (8.9%)	0.234	0.011
Nicotine dependence	1483 (6.2%)	22 193 (7.4%)	<0.0001	0.048	1483 (6.2%)	1385 (5.7%)	0.059	0.017
Personal history of nicotine dependence	1052 (4.4%)	49 714 (16.5%)	<0.0001	0.404	1052 (4.4%)	1020 (4.2%)	0.472	0.007
Sleep apnoea	1457 (6.0%)	30 507 (10.1%)	<0.0001	0.15	1457 (6.0%)	1340 (5.6%)	0.023	0.021
Other chronic obstructive pulmonary disease	769 (3.2%)	22 818 (7.6%)	<0.0001	0.195	769 (3.2%)	716 (3.0%)	0.162	0.013
Contact with and (suspected) exposure to asbestos	10 (0.0%)	436 (0.1%)	<0.0001	0.034	10 (0.0%)	10 (0.0%)	1	0
Pneumoconiosis due to dust containing silica	10 (0.0%)	17 (0.0%)	0.041	0.023	10 (0.0%)	0	0.002	0.029

Medications								

Nitrofurantoin	550 (2.3%)	7531 (2.5%)	0.012	0.014	550 (2.3%)	501 (2.1%)	0.126	0.014
Amiodarone	106 (0.4%)	10 103 (3.3%)	<0.0001	0.215	106 (0.4%)	115 (0.5%)	0.544	0.006

#### Post‐match comparisons

After matching, both cohorts retained 24 091 patients each. Standardized differences fell below 0.1, indicating a good balance across most covariates (Table 3).

#### Risk of ILD

Over the same 1‐day to 5‐year period, ILD incidence was 0.28% in PsO patients versus 0.35% in the matched general population. The RR was 0.79 (95% CI: 0.57–1.08; *P *= 0.141), suggesting no significant difference in ILD risk between PsO patients and controls (Table 2).

### PsA versus psoriasis

#### Baseline characteristics before matching

Before PSM, 19 741 PsA patients were compared with 24 616 PsO patients (Table 4). PsA patients tended to be slightly older (50.4 ± 14 vs. 47.6 ± 18 years, *P *< 0.001) and had a higher mean BMI (32.1 ± 7.7 vs. 30.8 ± 8.0, *P *< 0.001). They also had more frequent comorbidities (e.g. GERD, sleep apnoea) and higher use of cDMARDs and bDMARDS.

**Table 4.** Baseline characteristics of PsA versus psoriasis before and after propensity score matching

	Unadjusted baseline characteristics				Adjusted baseline characteristics			
	PsA	Psoriasis	*P*-value	Std diff.	PsA	Psoriasis	*P*-value	Std diff.
	(*n* = 19 741)	(*n* = 24 616)			(*n* = 13 873)	(*n* = 13 873)		
	Mean ± SD	Mean ± SD			Mean ± SD	Mean ± SD		
Age at index	50.4 ± 14	47.6 ± 18	<0.0001	0.1776	49.4 ± 13.9	50.4 ± 16.6	<0.0001	0.0605
BMI	32.1 ± 7.74	30.8 ± 8.02	<0.0001	0.1607	31.9 ± 7.65	31.2 ± 7.89	<0.0001	0.0913

	Unadjusted # patients (% of cohort)				Adjusted # patients (% of cohort)			

White	15 978 (80.90%)	17 881 (72.64%)	<0.0001	0.1975	10 981 (79.15%)	11 059 (79.72%)	0.2466	0.0139
Female	10 853 (54.98%)	12 027 (48.86%)	<0.0001	0.1227	7297 (52.60%)	7166 (51.65%)	0.1154	0.0189
Male	8199 (41.53%)	12 034 (48.89%)	<0.0001	0.1482	6006 (43.29%)	6446 (46.46%)	<0.0001	0.0638
Black or African American	571 (2.89%)	1525 (6.20%)	<0.0001	0.1591	452 (3.26%)	389 (2.80%)	0.0274	0.0265
Asian	624 (3.16%)	1156 (4.70%)	<0.0001	0.0791	482 (3.47%)	461 (3.32%)	0.4866	0.0084

Medical History	Unadjusted # patients (% of cohort)				Adjusted # patients (% of cohort)			

Gastro-esophageal reflux disease	3381 (17.13%)	3202 (13.01%)	<0.0001	0.1153	2132 (15.37%)	2033 (14.65%)	0.0961	0.0200
Sleep apnoea	2177 (11.03%)	2111 (8.58%)	<0.0001	0.0825	1333 (9.61%)	1304 (9.40%)	0.5527	0.0071
Nicotine dependence	1644 (8.33%)	2431 (9.88%)	<0.0001	0.0538	1241 (8.95%)	1178 (8.49%)	0.1800	0.0161
Asthma	1682 (8.52%)	1789 (7.27%)	<0.0001	0.0465	1148 (8.26%)	985 (7.10%)	0.0002	0.0441
Personal history of nicotine dependence	1270 (6.43%)	1627 (6.61%)	0.4554	0.0071	904 (6.52%)	860 (6.20%)	0.2790	0.0130
Fatty (change of) liver, not elsewhere classified	876 (4.44%)	828 (3.36%)	<0.0001	0.055	623 (4.49%)	462 (3.33%)	<0.0001	0.0599
Other chronic obstructive pulmonary disease	562 (2.85%)	963 (3.91%)	<0.0001	0.0590	433 (3.12%)	369 (2.66%)	0.0218	0.0275
Contact with and (suspected) exposure to asbestos	10 (0.05%)	11 (0.05%)	0.7739	0.0027	10 (0.07%)	10 (0.07%)	1	<0.0001
Pneumoconiosis due to dust containing silica	10 (0.05%)	10 (0.04%)	0.6209	0.0047	10 (0.07%)	10 (0.07%)	1	<0.0001
Contact with and (suspected) exposure to mold (toxic)	10 (0.05%)	10 (0.04%)	0.6209	0.0047	10 (0.07%)	10 (0.07%)	1	<0.0001
Occupational exposure to dust	0 (0%)	10 (0.041%)	0.0046	0.0285	0 (0%)	10 (0.072%)	0.0016	0.0380

Medications								

Tumour necrosis factor-alpha (TNF-alpha) inhibitors	8721 (44.18%)	7184 (29.18%)	<0.0001	0.3149	5414 (39.03%)	5523 (39.81%)	0.1805	0.0161
Methotrexate	7887 (39.95%)	5763 (23.41%)	<0.0001	0.3613	4599 (33.15%)	4700 (33.88%)	0.1990	0.0154
Apremilast	2744 (13.90%)	5263 (21.38)	<0.0001	0.1972	2089 (15.06%)	1932 (13.93%)	0.0074	0.0322
Ustekinumab	1273 (6.45%)	2956 (12.01%)	<0.0001	0.1930	957 (6.90%)	919 (6.62%)	0.3636	0.0109
Secukinumab	1824 (9.24%)	1361 (5.53%)	<0.0001	0.1423	1063 (7.66%)	1044 (7.53%)	0.6668	0.0052
Ixekizumab	918 (4.65%)	1232 (5.01%)	0.0839	0.0165	589 (4.25%)	523 (3.77%)	0.0434	0.0243
Nitrofurantoin	858 (4.35%)	991 (4.03%)	0.0933	0.0160	561 (4.04%)	511 (3.68%)	0.1194	0.0187
Guselkumab	534 (4.1%)	784 (3.3%)	<0.0001	0.043	399 (3.9%)	374 (3.6%)	0.359	0.013
Sulfasalazine	1729 (8.76%)	135 (0.55%)	<0.0001	0.3974	177 (1.28%)	135 (0.97%)	0.0168	0.0287
Leflunomide	1050 (5.32%)	88 (0.36%)	<0.0001	0.3022	132 (0.95%)	88 (0.63%)	0.0029	0.0358
Risankizumab	398 (2.02%)	451 (1.83%)	0.1599	0.0134	280 (2.02%)	315 (2.27%)	0.1469	0.0174
Janus-associated kinase (JAK) inhibitors	409 (2.07%)	99 (0.40%)	<0.0001	0.1515	108 (0.78%)	98 (0.71%)	0.4843	0.0084
Abatacept	121 (0.61%)	12 (0.05%)	<0.0001	0.0984	24 (0.17%)	12 (0.09%)	0.0454	0.0240
Amiodarone	69 (0.35%)	159 (0.65%)	<0.0001	0.0421	52 (0.36%)	43 (0.31%)	0.3550	0.0111
Brodalumab	27 (0.14%)	23 (0.09%)	0.1764	0.0128	15 (0.11%)	10 (0.07%)	0.3171	0.0120

Comparisons of ILD risk between PsA patients and controls (analysis A), PsO patients and controls (analysis B) and between PsA and PsO patients (analysis C). The presence of a diagnosis of ILD (identified by ICD-10 codes) within a 1-day to 5-year window following the index event (first-time diagnosis of psoriatic arthritis for PsA or psoriasis for PsO) was evaluated. Any patient with a prior ILD diagnosis before their respective index event was excluded from analysis.

#### Post‐match comparisons

Following PSM, each group included 13 873 patients (Table 4). Most baseline factors became well balanced. However, PsA patients maintained a slightly higher rate of fatty liver disease (4.49% vs. 3.33%, *P *< 0.001). Other matched variables, including the distribution of anti-psoriatic bDMARDs, were comparable.

#### Risk of ILD

During the 1‐day to 5‐year post‐index window, ILD was identified in 0.53% of PsA patients compared with 0.35% of PsO patients. The RR was 1.52 (95% CI: 1.06–2.20; *P *< 0.05), indicating a significantly higher risk of ILD among those with PsA versus PsO (Table 2).

#### Negative control analysis

Neither injury (RR: 0.98; 95% CI: 0.67–1.44; *P* = 0.9190) nor Tdap vaccination (RR: 1.03; 95% CI: 0.92–1.16; *P* = 0.5757) differed significantly between the PsA and PsO cohorts, supporting minimal residual confounding and reinforcing the robustness of our primary findings regarding increased ILD risk (Supplementary Table S3, available at *Rheumatology Advances in Practice* online).

## Discussion

To our knowledge, this is the largest-scale investigation of the risk of ILD in patients with PsA. Our findings indicate that PsA patients are significantly more likely to be diagnosed with ILD than PsO patients. Our results show that patients with PsA had a significantly increased risk of ILD compared with both controls (RR = 1.94; 95% CI [1.29–2.92]; *P* < 0.01) and patients with PsO (RR = 1.52; 95% CI [1.06–2.20]; *P* < 0.05). Patients with PsO and without PsA showed no increased ILD risk compared with controls (RR = 0.79; 95% CI [0.57–1.08]; *P* = 0.141).

Our findings align with case reports and studies documenting ILD associated with psoriasis and PsA [5–9, 12]. Our results largely mirror the results of the aforementioned 2024 Nordic observational study. That study also relied on the J84 ICD-10 code to establish the presence of ILD [15]. However, their definition of ILD also included patients with other ICD-10 codes (J70/respiratory conditions due to other external agents and J99/respiratory disorder in diseases classified elsewhere). Our results also align well with those of a recent Mendelian randomization study, which found a causal impact of PsA on ILD but did not find any connection between PsO and ILD [23].

Several pathways have been proposed to mediate the possible association between psoriasis and ILD. A 2022 systematic review highlighted common risk factors (e.g. smoking, depression, psoriasis medications), genetic factors, epigenetics and chronic inflammation as possible underlying mechanisms [24]. Despite ongoing and recent research investigating psoriasis and ILDs, a consensus mechanism has yet to be established.

Advanced age is a significant risk factor for ILD. Additionally, the initial manifestation of psoriasis typically occurs about 10 years before the development of joint disease [4]. As a result, PsA patients are generally older than patients with isolated skin disease. However, since our analysis accounted for age through PSM, the increased risk of ILD is not attributable to advanced age. BMI was a covariate in PSM in all three pairwise analyses, but PsA patients had a slightly higher BMI than the control cohort. Increased BMI has been identified as a risk factor for ILD (specifically IPF) by Mendelian randomization studies [25, 26]. However, cohort studies examining IPF patients found higher BMI paradoxically correlated with lower mortality and degree of fibrosis in this population [27–29]. Therefore, while BMI has been identified as a possible risk factor for ILD in Mendelian Randomization studies, the contrary findings in human cohort studies underscore the complex nature of the relationship and suggest that BMI is unlikely to confound our results. Potential confounding related to medications prescribed for PsA should also be considered. Case studies have documented occurrences of ILD associated with the use of leflunomide and SSZ in PsA patients [5, 6]. Several reports have similarly noted potential drug-induced ILD in psoriasis patients treated with ixekizumab and ustekinumab [30–33]. Methotrexate has historically been implicated in fibrotic ILD, although substantial evidence contradicts this association [34]. SSZ, specifically, was inherently more common in the PsA cohort compared with PsO, given its primary indication for inflammatory arthritis and lack of efficacy in psoriatic skin disease. Previous literature has indicated SSZ’s possible association with ILD [5, 35]. However, PSM in our analysis effectively balanced SSZ usage between cohorts (SMD <0.01), indicating excellent covariate balance. Nevertheless, residual confounding from medication use cannot be entirely excluded. Several case reports and smaller studies have additionally described associations between anti-TNF-alpha therapy and ILD [36–40], although larger cohort studies have not confirmed this finding [41]. To further mitigate confounding from medication use, we included the use of TNF-alpha inhibitors, ixekizumab, ustekinumab, methotrexate, JAK inhibitors, guselkumab and several other common psoriasis and PsA therapies as covariates in our PSM approach. A complete list of covariates included in the PSM is listed in Supplementary Table S2, available at *Rheumatology Advances in Practice* online.

Another mechanism underlying the increased association of PsA and ILD could be the presence of undetected antibodies in PsA patients. Antibodies to compounds present in the lung and extra-pulmonary sites have been implicated in rheumatoid arthritis-associated ILD [42]. Approximately 10% of PsA patients were seropositive for anti-CCP antibodies, with their presence associated with more severe phenotypes of PsA [43, 44]. In this study, we excluded patients with measured elevated levels of anti-CCP antibodies. However, the presence of anti-CCP antibodies in PsA patients who were not tested or the presence of an antibody targeting a yet-to-be-identified antigen could potentially explain the increased risk for ILD in PsA patients.

The strengths of this study include the large sample size and wide geographic distribution of the TriNetX research network. Previous studies have estimated the positive predictive value (PPV) of psoriasis and PsA diagnosis codes to be approximately 90% and 80%, respectively [45–47]. We also controlled for several possible confounding variables. Inclusion criteria for both PsO and PsA cohorts included the use of immunosuppressant medications, and exclusion criteria for both cohorts included a diagnosis of possible confounding factors such as sarcoidosis, ankylosing spondylitis and other conditions. These additional inclusion and exclusion criteria likely increased the PPV of the diagnosis codes used to describe psoriasis and PsA in this study. As previously stated, this study represents the largest analysis investigating the RR of PsA and PsO patients compared with each other and healthy controls.

This study does have limitations to consider. Healthcare organization EMR data such as the one used in this study are subject to entry errors, misdiagnosis and diagnoses occurring outside the data network would not be recorded. Given the rare nature of ILDs, this could possibly introduce bias. The retrospective and aggregated nature of the data also prevents a more granular analysis of the clinical criteria that led to a diagnosis of psoriasis, PsA or ILD. ILD pathological subtypes were also not reported, such as NSIP or UIP. This also meant we could not evaluate the diagnostic criteria used—such as HRCT scans or pulmonary function tests—to confirm and follow up each case of ILD. This study demonstrated that the risk of ILD seems to be elevated in PsA patients. However, this study did not examine the specific types of ILD for which these patients are at an increased risk beyond the general ILD code reported or determine the impact ILD had on patient outcomes. The absence of subtype-specific data prevents assessment of whether PsA-associated ILD aligns with known RA-ILD patterns (e.g. UIP predominance) or represents distinct entities. Future research is needed to further examine the epidemiological associations between psoriasis and ILD with a higher degree of granularity. Additional research is also needed to determine the effects of medication use, possible novel antibodies and inflammation to elucidate the pathways mediating the increased ILD risk found in this observational study.

In summary, our work provides compelling evidence that PsA is a risk factor for ILD, highlighting a clinically significant and previously underappreciated association. These findings not only raise awareness of ILD as a potential extra-articular manifestation of PsA but also underscore the importance of screening and monitoring for pulmonary involvement in this patient population. This study also lays the foundation for future research, including large-scale epidemiological investigations to better define risk factors and mechanistic studies aimed at uncovering the biological pathways that link PsA to ILD.

## Supplementary Material

rkaf059_Supplementary_Data

## Data Availability

The data underlying this study were obtained from the TriNetX US Collaborative Network, a federated EHR database that aggregates de-identified patient records from participating healthcare organizations. Due to TriNetX’s data use agreements and HIPAA compliance requirements, the raw data cannot be made publicly available. Researchers may request access to TriNetX through institutional agreements (https://www.trinetx.com/). Aggregated results supporting the findings are available from the corresponding author upon reasonable request.
