# Supplementary material for: The risk of interstitial lung disease in psoriatic arthritis versus psoriasis: a retrospective nationwide database analysis (2014–24)
Source: Rheumatol Adv Pract. 2025 May 27;9(3):rkaf059. doi: 10.1093/rap/rkaf059 (PMC12161981; doi:10.1093/rap/rkaf059)
Supplement: rkaf059_Supplementary_Data [file rkaf059_supplementary_data.docx]

| Supplementary Table S1: ICD-10 and RxNorm Codes distribution for the diagnoses, medications, and labs used in our analyses | |
| --- | --- |
| Code | **Diagnosis/Medication/Lab** |
| Rheumatological diseases | |
| UMLS:ICD10CM:L40 | Psoriasis |
| UMLS:ICD10CM:L40.50 | Arthropathic psoriasis |
| UMLS:ICD10CM:M06.9 | Rheumatoid arthritis, unspecified |
| UMLS:ICD10CM:L93 | Lupus erythematous |
| UMLS:ICD10CM:M32 | Systemic lupus erythematosus (SLE) |
| UMLS:ICD10CM:M33 | Dermatopolymyositis |
| UMLS:ICD10CM:M31.6 | Other giant cell arteritis |
| UMLS:ICD10CM:I77.82 | Antineutrophilic cytoplasmic antibody {ANCA] vasculitis |
| UMLS:ICD10CM:M31.6 | Other giant cell arteritis |
| UMLS:ICD10CM:M35.3 | Polymyalgia rheumatica |
| UMLS:ICD10CM:M31.4 | Aortic arch syndrome [Takayasu] |
| UMLS:ICD10CM:I77.82 | Antineutrophilic cytoplasmic antibody [ANCA] vasculitis |
| UMLS:ICD10CM:M35.00 | Sjögren syndrome, unspecified |
| UMLS:ICD10CM:M35.3 | Polymyalgia rheumatica |
| UMLS:ICD10CM:M34 | Systemic sclerosis [scleroderma] |
| UMLS:ICD10CM:D89.84 | IgG4-related disease |
| UMLS:ICD10CM:Z94 | Transplanted organ and tissue status |
| UMLS:ICD10CM:C81-C96 | Malignant neoplasms of lymphoid, hematopoietic and related tissue |
| UMLS:ICD10CM:C80.1 | Malignant (primary) neoplasm, unspecified |
| UMLS:ICD10CM:M35 | Other systemic involvement of connective tissue |
| UMLS:ICD10CM:M45 | Ankylosing spondylitis |
| UMLS:ICD10CM:M06.4 | Inflammatory polyarthropathy |
| UMLS:ICD10CM:K50 | Crohn’s disease [regional enteritis] |
| UMLS:ICD10CM:K51 | Ulcerative colitis |
| UMLS:ICD10CM:L10.9 | Pemphigus, unspecified |
| UMLS:ICD10CM:L20.89 | Other atopic dermatitis |
| UMLS:ICD10CM:G35 | Multiple sclerosis |
| UMLS:ICD10CM:M30.1 | Polyarteritis with lung involvement [Churg-Strauss] |
| UMLS:ICD10CM:D86 | Sarcoidosis |
| UMLS:ICD10CM:M08.00 | Unspecified juvenile rheumatoid arthritis of unspecified site |
| UMLS:ICD10CM:M31.4 | Aortic arch syndrome [Takayasu] |
| UMLS:ICD10CM:L90.0 | Lichen sclerosus et atrophicus |
| UMLS:ICD10CM:L94.0 | Localized scleroderma [morphea] |
| UMLS:ICD10CM:L73.2 | Hidradenitis suppurativa |
| UMLS:ICD10CM:L30.0 | Nummular dermatitis |
| UMLS:ICD10CM:L20 | Atopic dermatitis |
| UMLS:ICD10CM:L21 | Seborrheic dermatitis |
| UMLS:ICD10CM:L28.0 | Lichen simplex chronicus |
| Immunosuppressive agents | |
| NLM:RXNORM:327361 | adalimumab |
| NLM:RXNORM:6851 | methotrexate |
| NLM:RXNORM:709271 | certolizumab pegol |
| NLM:RXNORM:214555 | etanercept |
| NLM:RXNORM:3008 | cyclosporine |
| NLM:RXNORM:1599788 | secukinumab |
| NLM:RXNORM:191831 | infliximab |
| NLM:RXNORM:847083 | ustekinumab |
| NLM:RXNORM:819300 | golimumab |
| NLM:RXNORM:2053436 | tildrakizumab |
| NLM:RXNORM:1928588 | guselkumab |
| NLM:RXNORM:1872251 | brodalumab |
| NLM:RXNORM:2612087 | deucravacitinib |
| NLM:RXNORM:1492727 | apremilast |
| NLM:RXNORM:2668041 | bimekizumab |
| NLM:RXNORM:1256 | azathioprine |
| NLM:RXNORM:68149 | mycophenolate mofetil |
| NLM:RXNORM:1745099 | ixekizumab |
| Interstitial lung diseases | |
| UMLS:ICD10CM:J84.9 | Interstitial pulmonary disease, unspecified |
| UMLS:ICD10CM:J84.10 | Pulmonary fibrosis, unspecified |
| UMLS:ICD10CM:J84.170 | Interstitial lung disease with progressive fibrotic phenotype in diseases classified elsewhere |
| UMLS:ICD10CM:J84.89 | Other specified interstitial pulmonary diseases |
| UMLS:ICD10CM:J84.112 | Idiopathic pulmonary fibrosis |

| **Supplementary Table S2: ICD-10 Codes distribution for Propensity Score Matching** | |
| --- | --- |
| **Code** | **Characteristic** |
| Demographics | |
| AI | Age at Index |
| LOINC:39156-5 | BMI |
| 2106-3 | White |
| F | Female |
| M | Male |
| 2054-5 | Black or African American |
| 2028-9 | Asian |
| Diagnoses | |
| UMLS:ICD10CM:I10 | Essential (primary) hypertension |
| UMLS:ICD10CM:E78.5 | Hyperlipidemia, unspecified |
| UMLS:ICD10CM:K21 | Gastro-esophageal reflux disease |
| UMLS:ICD10CM:E08-E13 | Diabetes mellitus |
| UMLS:ICD10CM:G47.3 | Sleep apnea |
| UMLS:ICD10CM:J45 | Asthma |
| UMLS:ICD10CM:F17 | Nicotine dependence |
| UMLS:ICD10CM:Z87.891 | Personal history of nicotine dependence |
| UMLS:ICD10CM:K76.0 | Fatty (change of) liver, not elsewhere classified |
| UMLS:ICD10CM:J44 | Other chronic obstructive pulmonary disease |
| UMLS:ICD10CM:Z77.090 | Contact with and (suspected) exposure to asbestos |
| UMLS:ICD10CM:J62 | Pneumoconiosis due to dust containing silica |

**Supplementary Table S3. Negative Control Analysis**

| **Sensitivity Analysis A (5 years)**  **Adjusted Risk Ratio**  *33 patients in Cohort 1 and 41 patients in Cohort 2 were excluded from results because they had the outcome prior to the time window.* | | | | |
| --- | --- | --- | --- | --- |
| Psoriatic arthritis (*n* = 13,840) | Psoriasis (*n* = 13,832) | *Risk ratio* | *p* value | Confidence interval (CI) |
| 51 (0.37%) | 52 (0.38%) | 0.98 | 0.92 | 0.67-1.44 |
| **Sensitivity Analysis B (5 years)**  **Adjusted Risk Ratio**  *731 patients in Cohort 1 and 965 patients in Cohort 2 were excluded from results because they had the outcome prior to the time window.* | | | | |
| Psoriatic arthritis (*n* = 13,142) | Psoriasis (*n* =12,908) | *Risk ratio* | *p* value* | Confidence interval (CI) |
| 593 (4.51%) | 564 (4.37%) | 1.033 | 0.58 | 0.92-1.16 |

Sensitivity analyses were performed using two negative control outcomes—injury/Sensitivity Analysis A (ICD-10 T14.90XA) and Tdap vaccination/Sensitivity Analysis B (CPT 90715)—to evaluate residual confounding. Negative controls were selected based on their presumed independence from ILD risk and PsA/PsO disease processes.
